# Supplementary material for: Development of a standardized MRI protocol for pancreas assessment in humans
Source: PLoS One. 2021 Aug 24;16(8):e0256029. doi: 10.1371/journal.pone.0256029 (PMC8384163; doi:10.1371/journal.pone.0256029)
Supplement: Attachment — Submitted filename: Response to Reviewers.docx [file pone.0256029.s001.docx]

June 21, 2021

Dear Dr. Jin:

Thank you for review of our manuscript entitled ‘Development of a Standardized MRI Protocol for Pancreas Assessment in Humans’ for consideration by the journal *PLOS ONE.*

We have revised our manuscript based upon the constructive criticism of the reviewers and believe that the revised manuscript is greatly improved. The revised manuscript is provided with both ‘tracked changes’ and a ‘clean’ version. We address each reviewer comment below (author response in italics):

**Journal Requirements:**

1. Please ensure that your manuscript meets PLOS ONE's style requirements, including those for file naming.

*Author Response: The manuscript has been reformatted according to PLOS ONE style guidelines.*

2. We note that you have stated that you will provide repository information for your data at acceptance. Should your manuscript be accepted for publication, we will hold it until you provide the relevant accession numbers or DOIs necessary to access your data. If you wish to make changes to your Data Availability statement, please describe these changes in your cover letter and we will update your Data Availability statement to reflect the information you provide.

*Author Response: We would like to alter our Data Availability statement to match that of our submitted manuscript: ‘The datasets generated during and/or analyzed during the current study are available from the corresponding author upon reasonable request. The image acquisition and processing protocols used in these studies are available online:* [*https://github.com/jvirostko/MAPT1D*](https://github.com/jvirostko/MAPT1D)*.’ This change has been noted in a revised cover letter accompanying this resubmission.*

3. We note that Figure 1 in your submission contain map images which may be copyrighted… If you are unable to obtain permission from the original copyright holder to publish these figures under the CC BY 4.0 license or if the copyright holder’s requirements are incompatible with the CC BY 4.0 license, please either i) remove the figure or ii) supply a replacement figure that complies with the CC BY 4.0 license.

*Author Response: The map in Figure 1B has been replaced with a US map generated by our group.*

**Reviewer #1:**

1. Since obesity and NAFLD are risk factors in the development of diabetes, evaluation of adipose tissue and the liver would strengthen this protocol.

*Author Response: We agree with the reviewer and respectfully note that we have included liver fat analysis in the current manuscript. However, in order to focus on pancreas imaging, which is currently less developed than liver imaging, we have chosen to limit the studies of liver imaging in this work. Liver and adipose tissue reproducibility will be analyzed in future studies.*

2. It has been well-established that liver stiffness can be a reliable alternative to biopsy for assessing hepatic fibrosis and providing prognostic value as well. There are many publications about pancreatic MRE from Dr. Yu Shi in this field. The author could make a complete imaging characterization by adding this imaging method.

*Author Response: We have added a citation to Dr. Shi’s work and MRE to the discussion section: ‘Magnetic resonance elastography is another promising technique for assessing the pancreas, but as it requires specialized hardware and software not available at our study sites it was not included in this study. However, a previous study examining the reproducibility of magnetic resonance elastography in healthy controls indicates that it has similar reproducibility to the T1 and ADC measurements in this study (39).’*

3. Methods: what’s the time range for phantom scan between locations? According to Ref 23, there was little change in a longitudinal study of 25days for the ice water phantom, but no mention of longer time. In Ref 24, they used 5% and 10% BSA, no mention of 15%, or any dynamic changes. Will the reference value change during time? If not, any other reference?

*Author Response: All phantoms were scanned within 60 days of each other, and we did not detect any differences over the timescale of these studies. This has been added to the methods and results section.*

4. Methods: how big are the ROIs for calculating fat fraction, pancreas and liver, respectively? And where are the ROIs located, since the fat fraction varies much between locations.

*Author Response: We apologize for the lack of clarity and have edited the Methods section to provide these details as follows: ‘For each multiparametric MRI map (ADC, T1, MTR, fat fraction), the pancreas volume outlined by the radiologist was co-registered and re-gridded to the image resolution of each parametric map to generate a whole-pancreas region of interest. Each MRI parameter was then averaged throughout the whole-pancreas region of interest to yield the mean value of each parameter for voxels within the pancreas. For hepatic fat fraction a circular region of interest of area 4 cm2 was placed on a single slice of the right lobe of the liver while carefully avoiding large vessels and bile ducts.’*

5. Results: the hepatic fat fraction of Volunteers 5, is over 5%, which is diagnosed as fatty liver. In that case, it might influence the measurements of pancreas.

*Author Response: We agree with the reviewer that this is an important finding and have added the association between liver and pancreas fat to the discussion: ‘. For example, one individual (denoted as Volunteer 5 in Table 2) had increased fat content in both the liver and pancreas. An association between fat accumulation the liver and pancreas has been previously reported (36).’*

6. Discussion: the measurements of fat fraction are all missing in Denver, is there any reason?

*Author Response: The scanner in Denver did not have software required for acquiring fat fraction maps as noted in the Methods: ‘A 3D quantitative 6-point Dixon acquisition was collected at sites which had the requisite software on their scanner (Austin, Nashville, Chicago, Melbourne)’. This is also noted in the legend for Figure 3: ‘Note that fat fraction was not measured in Denver due to a lack of the requisite software.’*

7. Figure 1: the figure and descriptions in manuscript should be consistent. eg, in the manuscript Methods_Phantom Studies, it was described as T1, MTR, and fat fraction, in the figure it showed “T1/MTR/Fat” in the top row , but the bottom row indicate Canola oil, BSA, and Gadolinium, which are used for fat fraction, MTR, and T1, respectively. Besides, it is also really confusing with three same color pattern and no unit or label at all.

*Author Response: We apologize for this inconsistency. We have relabeled the panels in Figure 1 to remain consistent and added units to each colorbar.*

8. Figure 4A, please indicate all the measurements, it is difficult to tell changes from the color.

*Author Response: Mean pancreas values for T1 and ADC have been added to each panel of Figure 4A to aid interpretation of these figures.*

**Reviewer #2:**

1. In MRI Scanning Protocol section, list in a table of scanner vendors, model and software version will be helpful for understanding the results.

*Author Response: We thank the reviewer for catching our omission of scanner software. We have added it along with the description of the scanner vendor and models to the first paragraph of the methods section: ‘Two centers (Austin and Denver) were equipped with 3T Skyra scanners (Siemens, Erlangen, Germany). Scans performed in Melbourne were acquired using a 3T Prisma scanner (Siemens, Erlangen, Germany). Two centers (Nashville and Chicago) acquired images on 3T Ingenia scanners (Philips, Best, Netherlands). All Siemens scanners employed VE11C software. Of the Philips scanners, Vanderbilt employed R5.5.0.1 while Chicago employed R.5.6.1.’ As there are a number of repeated values (e.g., all Siemens sites use the same software, we believe that another table is not needed to present these parameters.*

2. In Table 1, can it include the bandwidth? b vectors for DWI? Regarding to T1 mapping, what does “5 acquisitions: 5 flip angles”? 5 series?

*Author Response: The b-values for DWI are provided in the ‘Miscellaneous’ row under the ‘DWI’ column in Table 1. We apologize for the confusion in the T1 mapping column. We have rewritten this to ‘5 flip angles’ to limit reader confusion. Bandwidth was not standardized across all scanners due to differences in sequence parameters and thus is not included in Table 1.*

3. In table 2, it seems the quantitative T1 values in via ,2,3,4 in Melbourne is lower than these in other sites? Is it the system bias?

*Author Response: We have added the possibility of bias to the results section: ‘Of note, one site (Melbourne) had the lowest T1 measurement for each phantom, suggesting there may be bias in T1 mapping on this scanner.’*

4. Could you please comment on that if we can normalize the data with standardized phantom data for the quantitative measurements in humans/individuals?

*Author Response: This is a really interesting idea. Unfortunately, preliminary standardization based on phantom results do not improve reproducibility of human scans. This may stem in part from the fact that the phantom scans were largely reproducible. Unfortunately, human scans were not performed in Melbourne due to travel cost concerns. As the reviewer astutely previously noted, there may be bias in T1 measurement in Melbourne which may be improved by standardizing to phantom bias.*

5. How many radiologists were involved in for outlining pancreas volume? Were the same radiologists involved in doing the same work for all sites’ studies? If not, any bias?

*Author Response: A single radiologist outlined all pancreas images as described in the Methods section: ‘The pancreas was outlined on each slice of the fat-suppressed T2-weighted image by an experienced radiologist (M.A.H.).’ We have added this use of a single reader as a limitation of this study to the Discussion section: ‘An additional limitation of this study is the use of a single reader to outline all pancreas images in this study. Multiple readers may lead to variation in the MRI measurements performed in this study (29).’*

6. More volunteer’s data will be better.

*Author Response: We agree with the reviewer that more subjects would increase the power of this work. Unfortunately, the cost of flying each volunteer to three separate locations limited the current study to 5 volunteers each scanned at 4 locations. We note this limitation in the Discussion section of this manuscript: ‘Finally, a larger volunteer cohort would allow for a more precise evaluation of variability. Sending the same individual to each study site for scanning is both expensive and technically difficult, and thus, it is not commonly performed. Given the complexity of navigating volunteer travel and scheduling, we were limited to five individuals scanned at four different sites but showed good agreement in MRI measurements across a wide variety of MRI hardware and software.’ We also note that our previous study of breast MRI reproducibility included only 3 volunteers due to the logistical difficulty of these multisite MRI studies (J Magn Reson Imaging. : 10.1002/jmri.26011.)*

Thank you for your input into this work. Please address all correspondence concerning this manuscript to me at [jack.virostko@austin.utexas.edu](mailto:jack.virostko@austin.utexas.edu).

Sincerely,

John (Jack) Virostko

512-495-5126

jack.virostko@austin.utexas.edu
